# Etiology of Anemia and Risk Factors of Mortality among Hospitalized Patients: A Real-Life Retrospective Study in a Tertiary Center in Greece

**DOI:** 10.3390/hematolrep15020036

**Published:** 2023-06-02

**Authors:** Petros Ioannou, Andria Papazachariou, Maria Tsafaridou, Ioannis E. Koutroubakis, Diamantis P. Kofteridis

**Affiliations:** 1 School of Medicine, University of Crete, 71003 Heraklion, Greece; 2 Internal Medicine Department, University Hospital of Heraklion, 71110 Heraklion, Greece; 3 Gastroenterology Department, University Hospital of Heraklion, 71110 Heraklion, Greece

**Keywords:** anemia, hemoglobin, hematocrit, blood transfusion

## Abstract

Anemia is a prominent global health issue with a wide variety of causes and can be associated with decreased quality of life, increased hospitalization, and higher mortality, especially in older individuals. Therefore, studies further shedding light on the causes and the risk factors of this condition should be performed. The aim of the present study was to examine the causes of anemia in hospitalized patients in a tertiary hospital in Greece and identify risk factors related to higher mortality. In total, 846 adult patients with a diagnosis of anemia were admitted during the study period. The median age was 81 years, and 44.8% were male. The majority of patients had microcytic anemia, with the median mean corpuscular volume (MCV) being 76.3 fL and the median hemoglobin being 7.1 g/dL. Antiplatelets were used by 28.6% of patients, while 28.4% were using anticoagulants at the time of diagnosis. At least one unit of packed red blood cells (PRBCs) was transfused in 84.6% of patients, and a median of two PRBCs was used per patient. A gastroscopy was performed in 55%, and a colonoscopy was performed in 39.8% of patients in the present cohort. Anemia was considered to be multifactorial in almost half the cases, while the most commonly identified cause was iron deficiency anemia, more commonly with positive endoscopic findings. Mortality was relatively low, at 4.1%. Multivariate logistic regression analysis identified higher B12 levels and longer duration of hospital stay to be independently positively associated with mortality.

## 1. Introduction

Anemia is a prominent global health issue with a wide variety of causes [1]. It may affect a substantial number of older individuals and is associated with increased morbidity and mortality, especially over the age of 85 years [2]. Despite vigorous efforts to achieve sufficient primary prevention worldwide through iron supplementation and endoscopic interventions, its prevalence is on the rise [3].

According to the World Health Organization (WHO), anemia is defined as a reduction in hemoglobin concentration below a specific cut-point that depends on gender, age, ethnicity, and physiological status. Up until now, the universally accepted cut-off points in adults are Hb lower than 13 g/dL in men and lower than 12 g/dL in women [4]. Patients may be asymptomatic or may present with symptoms related to an underlying disease (such as cardiovascular disease), with an increased risk of falls, cognitive impairment, or decreased physical performance [5].

Numerous studies identify iron deficiency as the most common cause of anemia in the elderly, especially after the broad use of novel oral anticoagulants in people with atrial fibrillation, pulmonary embolism, or venous thromboembolism [6]. Besides iron deficiency, dietary insufficiency (such as that related to vitamin B12 deficiency or folate deficiency), anemia of chronic disease, chronic kidney disease (CKD)-associated anemia, and hematologic disorders are additionally common causes of anemia [7]. It is noteworthy that in up to one-third of older individuals, anemia may remain unexplained [8].

Anemia is associated with decreased quality of life, increased hospitalization, and higher mortality, especially in older individuals; thus, studies further shedding light on the causes and the risk factors of this condition should be performed [9]. The aim of the present study was to examine the causes of anemia in hospitalized patients in a tertiary hospital in Greece and identify risk factors related to higher mortality.

## 2. Materials and Methods

### 2.1. Study Population

This is a retrospective cohort study. The study participants were patients admitted to the Internal Medicine Department of the University Hospital of Heraklion, Crete, Greece, from April 2016 to September 2022. All adult patients who were admitted to this Department with a diagnosis of anemia were included. Data recorded and evaluated included age, gender, past medical history (heart failure, coronary artery disease, atrial fibrillation, cerebrovascular disease, hypertension, peripheral artery disease, diabetes mellitus, chronic kidney disease), medication use (NSAIDs, antiplatelet, anticoagulant, proton pump inhibitor), laboratory exams, endoscopic findings from gastroscopy and colonoscopy (when performed), treatment and transfusion of packed red blood cells, duration of hospitalization, and outcome. Microcytic anemia was defined as anemia with the presence of hypochromic small red blood cells in a peripheral blood smear or with the presence of low MCV (below 83 fL) [10]. Iron deficiency was defined as anemia with a ferritin concentration of less than 15 ng/mL [11]. Anemia was defined according to the World Health Organization (WHO) criteria that include a hemoglobin of less than 13 g/dL for men and less than 12 g/dL for women, even though patients admitted to the hospital had significantly lower than that [12].

The study was approved by the Ethics Committee of the University Hospital of Heraklion.

### 2.2. Statistics

Categorical data were analyzed with Fisher’s exact test. Continuous variables were compared using Student’s *t*-test for normally distributed variables and the Mann–Whitney U-test for non-normally distributed variables. All tests were two-tailed, and *p* ≤ 0.05 were considered to be significant. Data are presented as numbers (%) for categorical variables and medians [interquartile range (IQR)] or means [±standard deviation (SD)] for continuous variables. A linear regression analysis model was developed to evaluate the effect of several parameters (age, gender, past medical history, medication use, laboratory exams, duration of hospitalization, and treatment administered) on mortality. All the parameters mentioned above were calculated with GraphPad Prism 6.0 (GraphPad Software, Inc., San Diego, CA, USA). A multivariate logistic regression analysis model was developed to evaluate the association of factors identified in the univariate analysis with a *p* ≤ 0.05 with mortality. Multivariate analysis was performed using the SPSS version 23.0 (IBM Corp., Armonk, NY, USA).

## 3. Results

In total, 846 adult patients with a diagnosis of anemia were admitted to the Internal Medicine Department during the study period. Their epidemiological, clinical, and laboratory characteristics were recorded and evaluated. Table 1 shows the characteristics of the included patients as well as a comparison between male and female patients. The median age was 81 years (range 19–98 years), and 44.8% (379 patients) were male. The most common conditions from the past medical history were hypertension, heart failure, and chronic kidney disease. The majority of patients had microcytic anemia, with the median mean corpuscular volume (MCV) being 76.3 fL and the median hemoglobin being 7.1 g/dL. Antiplatelets were used by 28.6% of patients, while 28.4% were using anticoagulants at the time of diagnosis. At least one unit of packed red blood cells (PRBCs) was transfused in 84.6% of patients, and a median of two PRBCs was used per patient. A gastroscopy was performed in 55%, and a colonoscopy was performed in 39.8% of patients in the present cohort. Anemia was considered to be multifactorial in almost half the cases, while the most commonly identified cause was iron deficiency anemia, more commonly with positive endoscopic findings. Mortality was relatively low, at 4.1%. The findings during endoscopy are shown in Table 2.

Male patients were more likely to have coronary heart disease and peripheral artery disease in their past medical history, along with slightly higher creatinine on discharge. Male patients were also more likely to have higher hemoglobin on admission with a slightly higher mean corpuscular hemoglobin (MCH), MCV, mean corpuscular hemoglobin concentration (MCHC), and ferritin, while they had lower platelets and thyroid stimulating hormone (TSH). Male patients were more commonly on an antiplatelet agent on admission and were more likely to undergo colonoscopy, even though iron-deficiency anemia was more common among female patients. CKD-associated anemia was more common among female patients, but a hematological disease was the reason for anemia being more common among men. Mortality was similar among male and female patients.

Table 3 shows a comparison of characteristics among patients who survived with those who died. There was a slight trend for a higher age among patients who died, even though it did not reach statistical significance. History of CKD was more common among patients who died. Troponin I was higher in patients who died both on admission as well as in terms of its maximum value during hospitalization. MCV and MCH were higher among patients who died, and so were the white blood cells (WBCs). Vitamin B12 and ferritin were also higher among patients who died. More PRBCs were transfused to patients who died eventually, while gastroscopy and colonoscopy were more rarely used in this patient subpopulation. Iron deficiency was less common among patients who died, while CKD-associated anemia was more frequent. Finally, the duration of hospital stay was longer in those who died.

A regression analysis model was used to identify factors associated with mortality. More specifically, a univariate linear regression analysis model identified age, history of CKD, levels of troponin I on admission and maximum levels of troponin I during the hospital stay, MCH on admission, B12 levels, diagnosis of CKD-associated anemia, and duration of hospital stay to be positively associated with mortality and diagnosis of iron deficiency anemia to be negatively associated with mortality. However, a multivariate logistic regression analysis model identified only higher B12 levels and longer duration of hospital stay to be independently positively associated with mortality. The results of the regression analysis are shown in Table 4.

## 4. Discussion

This retrospective cohort study evaluated patients admitted to the Internal Medicine Department of a University Hospital with a diagnosis of anemia and identified a low mortality rate. In almost half the cases, the anemia was considered multifactorial, and the most commonly identified cause was iron-deficiency anemia, more commonly with positive endoscopic findings. Multivariate logistic regression analysis identified higher B12 levels and longer duration of hospital stay to be independently positively associated with mortality.

Anemia is one of the most frequent problems encountered in everyday clinical practice. In many patients that are otherwise healthy, the evaluation may be relatively easy and straightforward, however, in many patients, especially if a complex medical history is present, the diagnostic evaluation may be more complicated. Internationally proposed criteria for the diagnosis of anemia include those of the WHO that include a hemoglobin of less than 13 g/dL for men and less than 12 g/dL for women, even though these criteria are mainly intended to be used for international studies on nutrition and were not intended to be used clinically for the diagnosis of anemia in individual patients [12,13]. However, the reference ranges should not be seen as obsolete since normal ranges for laboratory tests are generally defined based on a range resulting from 95% of an otherwise healthy population. On the other hand, in specific populations, there may be normal variations affecting the definition of what may be considered normal and what should be considered anemia, as is the case with African Americans in whom lower hemoglobin of 0.5–1 g/dL may be considered normal [13,14,15,16,17]. However, it is not completely clear why this difference occurs and if it is the result of hemoglobinopathy, higher rates of iron deficiency anemia, or if it may be related to other causes [18].

In the present study, 846 patients were included over a period of about six years. The mean age was about 80 years, implying that the vast majority of the patients included were elderly. Older patients are known to suffer from anemia more commonly than young patients. For example, in another study involving 435 hospitalized patients admitted to the Internal Medicine Department of a hub hospital in Italy, 44% of them were identified as being anemic [19]. More than two-thirds of the patients in this cohort were older than 65 years old. Another study from a medical ward in Israel that compared the characteristics among anemic patients with and without diabetes also found that the majority of anemic patients were older than 65 years old [20]. Another study in a hospital in the United Kingdom found that among all patients that were admitted to medical and surgical wards, about half of them had anemia, and, again, the majority of these patients were elderly [21]. In the present study, the majority of patients were female. In the Italian study, there were more male patients compared to females [19], however, in the study from Israel, more female patients were diagnosed with anemia [20]. Large data from the Global Burden of Disease Study showed that there is an overall female predominance in the occurrence of anemia in 204 countries that provided data, even though these data do not refer only to inpatients [22].

Patients with anemia in the present study had cardiovascular comorbidities very often, as the vast majority had heart failure, coronary heart disease, or hypertension, while chronic kidney disease was also common. These findings were also noted in other studies as most of the patients had chronic cardiac disease, such as heart failure or coronary artery disease, and renal impairment was also quite frequent [20,21]. These findings are rather expected since these comorbidities are increasingly frequent with increased age, and as the majority of the patients admitted with anemia were elderly, an increasing prevalence of comorbidities was anticipated. As expected, antiplatelet use was more common among male patients with anemia since coronary artery disease was more common among this patient population. Interestingly, iron deficiency anemia was more common among female patients, despite the less frequent use of antiplatelet medications and a similar rate of anticoagulant use. Similarly, ferritin levels were higher in male patients than in female patients. However, according to data from 204 countries providing information to the Global Burden of Disease Study, it seems that in Eastern Europe, iron deficiency was a diagnosis more commonly made among male than female patients [22]. It is of note that the present study refers to inpatients only, while the study providing data from the Global Burden of Disease provides information from population-based surveys; thus, these two studies are not directly comparable since patients requiring hospitalization are more likely to have more severe anemia that may require hospitalization [22].

Among patients that underwent endoscopy, a gastroscopy was more frequently performed, followed by a colonoscopy. The most common findings in gastroscopy and colonoscopy identified underlying diagnoses that were indicative of bleeding that could explain iron-deficiency anemia. The value of endoscopy in the evaluation of iron-deficiency anemia is widely accepted both in premenopausal women as well as in patients over the age of 50 [23,24,25,26,27]. Indeed, beyond classic causes of iron-deficiency anemia, such as peptic ulcer disease and gastric or colonic cancer, recent studies shed light on other conditions of the gastrointestinal tract that could also be associated with iron-deficiency anemia, such as *Helicobacter pylori* infection or celiac disease [28,29]. However, some of the causes identified in the present study, such as polyps or fungal esophagitis, may not suffice to explain the anemia.

Overall in-hospital mortality was 4.1%, which is a rate lower than the one noted in all patients admitted in the same Internal Medicine Department in our hospital, as shown in another study that summarizes the characteristics of all admissions of patients hospitalized from 2013 until the end of 2019 [30]. Thus, patients with anemia may have a better prognosis; however, this study did not include data on patients’ follow-up, and mortality could be higher in the months following hospitalization. For example, in another study where follow-up of patients was provided for a median of 19 months, 17.3% of patients with anemia died [20]. Thus, it is important to acknowledge that patients with anemia may have significant underlying diseases that could be associated with the diagnosis of anemia which could increase mortality after initial diagnosis and hospitalization. To that end, we performed a comparison between patients who survived and those who died during their hospitalization for anemia. Patients that died had a non-significant trend for a higher age, while a history of CKD and the diagnosis of CKD-associated anemia were more common. These patients had higher MCV, MCH, and ferritin, while the diagnosis of iron deficiency anemia was less frequent compared to the group that survived. Thus, it may be reasonable that patients that died less frequently underwent gastroscopy and colonoscopy. On the other hand, the higher rate of death may imply a worse clinical condition of these patients that would not allow performing endoscopy during hospitalization. Finally, the duration of hospital stay was longer in those who died, which is generally a finding frequently noted in medical wards and noted in the previous study performed in our institution [30]. More specifically, in the present study, a multivariate logistic regression analysis identified an increased duration of stay to be independently associated with in-hospital mortality.

Interestingly, the same multivariate logistic regression analysis model identified high vitamin B12 to be independently associated with increased mortality among patients hospitalized with anemia. Indeed, there are studies in the literature suggesting an association between increased levels of plasma vitamin B12 levels and all-cause mortality in the general population [31]. Importantly, in this study in the Netherlands, this association of increased vitamin B12 and mortality persisted after adjusting for gender, renal function, age, and other clinical and laboratory parameters, even though the underlying pathophysiological association remains unclear [31]. One proposed mechanism is that high levels of vitamin B12 in the plasma could imply an increased release of vitamin B12 from the liver, decreased clearance, upregulation of biosynthesis, or reduced affinity of the vitamin for transporter proteins [32,33]. However, a definite explanation has not been described to date [34,35]. Of note, a similar association between increased levels of vitamin B12 and mortality has been identified in different patient populations, as well as in hemodialysis patients, the elderly, and patients with diabetes [36,37,38].

This study has some notable limitations. First of all, even though the sample is relatively large, it is a single-center study in an area that also has a relatively high rate of hemoglobinopathies (thalassemia), meaning that the results should be read cautiously, as they could be more applicable to similar patient populations. Larger multicenter studies could allow the drawing of safer conclusions. Furthermore, patients’ follow-up post-hospital discharge was not performed; thus, data on admissions or mortality after discharge were not available. Third, as data on follow-up were not available, data regarding endoscopy performed later on after discharge in patients who survived were also not available, implying that the data presented herein regarding endoscopy may not be reflective of the data referring to all endoscopies performed in total. Finally, the use of iron (oral or parenteral), folate, and B12 supplementation was not evaluated in the present study.

## 5. Conclusions

The present study presented 846 patients admitted with anemia to an Internal Medicine Department of a University Hospital in Greece. There was a female predominance, while the majority of patients were elderly. Differences were noted among male and female patients with anemia, mostly related to different comorbidities and treatments, while iron-deficiency anemia was less frequent among male patients. Gastroscopy and colonoscopy were performed in the majority of patients, with endoscopic findings justifying the diagnosis of iron deficiency in many cases. Mortality was relatively low. Duration of hospital stay and high levels of vitamin B12 were independently associated with increased in-hospital mortality. Future studies with larger and more diverse populations are needed to further investigate the epidemiology, pathophysiology, and management of anemia in different settings and populations.

## Figures and Tables

**Table 1:** Patients’ characteristics in total and regards to gender.

Characteristic	All Patients (*n* = 846)	Male Patients (*n* = 379)	Female Patients (*n* = 467)	*p*
Age, median (IQR)	81 (71–87)	80 (72–86)	82 (71–87)	0.39
Male gender, *n* (%)	379 (44.8)			
History of heart failure, *n* (%)	474 (56.2)	221 (58.5)	253 (54.4)	0.2641
Coronary heart disease, *n* (%)	252 (29.8)	155 (40.9)	97 (20.8)	<0.0001
Atrial fibrillation, *n* (%)	245 (29)	106 (28)	139 (29.8)	0.5938
Cerebrovascular disease, *n* (%)	81 (9.6)	44 (11.6)	37 (8)	0.0787
Hypertension, *n* (%)	612 (72.4)	274 (72.3)	338 (72.5)	0.9385
Peripheral artery disease, *n* (%)	63 (7.5)	39 (10.3)	24 (5.2)	0.0055
Diabetes mellitus, *n* (%)	276 (32.7)	135 (35.6)	141 (30.3)	0.1049
History of CKD, *n* (%)	465 (55)	195 (51.5)	270 (57.8)	0.0709
Creatinine on discharge, mg/dL, median (IQR)	1.1 (0.9–1.4)	1.2 (1–1.5)	1 (0.8–1.3)	<0.0001
Troponin I on admission, per ng/mL, median (IQR)	9.8 (3.6–24.8)	9.7 (3.6–32.5)	9.8 (3.7–23)	0.8705
Maximum troponin I during hospital stay, ng/mL, median (IQR)	11.3 (4.1–34.7)	11 (4–38.5)	11.9 (4.2–28.2)	0.8305
Hemoglobin on admission, g/dL, median (IQR)	7.1 (6.3–7.8)	7.3 (6.5–7.9)	6.9 (6.2–7.7)	<0.0001
MCV on admission, fL, median (IQR)	76.3 (66.2–87.1)	76.8 (67.4–88.5)	75.7 (65.1–85.4)	0.0274
MCH on admission, (pg), median (IQR)	23.9 (20.1–28.3)	24.6 (20.6–29)	23.5 (19.6–27.6)	0.0065
MCHC on admission, g/dL, median (IQR)	31.2 (30–32.4)	31.5 (30.2–32.7)	31 (29.9–32.2)	0.0012
RDW, %, median (IQR)	19.5 (17.2–22.3)	19.5 (17.1–21.8)	19.5 (17.3–22.7)	0.1398
WBCs, K/μL, median (IQR)	7.1 (5.4–9.5)	7.2 (5.3–9.2)	7 (5.4–9.7)	0.4635
Platelets, K/μL, median (IQR)	253 (181–335)	230 (171–313)	270 (189.8–355.3)	0.0004
TSH, mU/L, median (IQR)	1.4 (0.8–2.1)	1.3 (0.8–1.9)	1.4 (0.8–2.2)	0.0407
Vitamin B12, pg/mL, median (IQR)	364 (248–583)	358 (247–583)	365 (248.5–583.5)	0.9082
Folic acid, ng/mL, median (IQR)	6.8 (4.3–11.7)	6.3 (4.1–10.8)	7.1 (4.3–13.2)	0.064
Ferritin, μg/L, median (IQR)	22.4 (7.2–139.6)	25.4 (9–195.6)	20 (6.6–105.1)	0.0019
Antiplatelet use, *n* (%)	242 (28.6)	139 (36.7)	103 (22.1)	<0.0001
Anticoagulant use, *n* (%)	240 (28.4)	106 (28)	134 (28.8)	0.8184
Proton-pump inhibitor, *n* (%)	377 (44.7)	171 (45.1)	206 (44.3)	0.8348
Patients transfused, *n* (%)	715 (84.6)	312 (82.3)	403 (86.5)	0.1035
Number of PRBCs per person transfused, median (IQR)	2 (1–3)	2 (1–3)	2 (1–3)	0.0167
Gastroscopy performed, *n* (%)	465 (55)	216 (57)	249 (53.3)	0.3306
Colonoscopy performed, *n* (%)	337 (39.8)	169 (44.6)	168 (36)	0.0135
Diagnosis of anemia				
Multifactorial, *n* (%)	419 (49.6)	174 (46)	245 (52.5)	0.0720
Iron deficiency, *n* (%)	493 (58.3)	206 (54.4)	287 (61.5)	0.042
Vitamin B12 deficiency, *n* (%)	119 (14.1)	52 (13.7)	67 (14.3)	0.8426
Folate deficiency, *n* (%)	28 (3.3)	10 (2.6)	18 (3.9)	0.3426
Hematologic disease, *n* (%)	93 (11)	55 (14.5)	38 (8.1)	0.039
Gastrointestinal loss **, *n* (%)	375 (68.8)	185 (72.3)	190 (65.7)	0.1153
Urogenital blood loss, *n* (%)	61 (7.2)	20 (5.30)	41 (8.8)	0.0608
CKD-associated anemia, *n* (%)	369 (43.6)	143 (37.7)	226 (48.3)	0.0021
Hemolysis, *n* (%)	31 (3.7)	11 (2.9)	20 (4.3)	0.3585
Hemoglobinopathies, *n* (%)	55 (6.5)	21 (5.5)	34 (7.3)	0.3293
Other *, *n* (%)	80 (9.5)	37 (9.8)	43 (9.2)	0.8138
Duration of stay, days, median (IQR)	4 (2–6)	4 (2–7)	4 (2–6)	0.908
In-hospital mortality, *n* (%)	35 (4.1)	18 (4.7)	17 (3.6)	0.4885

* Metastatic malignancy, cirrhosis, heart failure, hypothyroidism, drug-induced. ** Among patients where endoscopy was performed. CKD: chronic kidney disease; IQR: interquartile range; MCH: mean corpuscular hemoglobin; MCHC: mean corpuscular hemoglobin concentration; MCV: mean corpuscular volume; OR: odds ratio; RDW: red cell distribution width; TSH: thyroid stimulating hormone; WBCs: white blood cells.

**Table 2:** Findings in endoscopy among patients admitted with anemia.

Gastroscopy Findings (in 465 Patients)	All Patients, *n* (%)	Colonoscopy Findings (in 336 Patients)	All Patients, *n* (%)
Erosive gastritis	89 (19.1)	Diverticula	58 (17.3)
Angioectasias	82 (17.6)	Colorectal cancer	53 (15.8)
Peptic ulcer	42 (9)	Angioectasias	51 (15.2)
Atrophic gastritis	33 (7.1)	Hemorrhoid disease	40 (11.9)
Cancer	21 (4.5)	Polyps	22 (6.5)
Esophagitis	17 (3.7)	Ischemic colitis	13 (3.9)
Esophageal varices	16 (3.4)	Ulcerations (colonic)	3 (0.9)
Ulcerations (gastric)	11 (2.7)	Anal fissure	2 (0.6)
Portal hypertensive gastropathy	11 (2.7)		
Polyps	5 (1.1)		
Duodenitis	3 (0.6)		
Fungal esophagitis	1 (0.2)		

**Table 3:** Patients’ characteristics in regards to mortality.

Characteristic	Survived (*n* = 811)	Died (*n* = 35)	*p*
Age, median (IQR)	81 (71–87)	84 (77–88)	0.0503
Male gender, *n* (%)	361 (44.5)	18 (51.4)	0.4885
History of heart failure, *n* (%)	453 (56)	21 (61.8)	0.5978
Coronary heart disease, *n* (%)	244 (30.1)	8 (23.5)	0.566
Atrial fibrillation, *n* (%)	235 (29)	10 (29.4)	1
Cerebrovascular disease, *n* (%)	76 (9.4)	5 (14.7)	0.3641
Hypertension, *n* (%)	586 (72.3)	26 (74.5)	0.6975
Peripheral artery disease, *n* (%)	62 (7.6)	1 (2.9)	0.5055
Diabetes mellitus, *n* (%)	266 (32.8)	10 (29.4)	0.8521
History of CKD, *n* (%)	435 (53.6)	30 (85.7)	0.0002
Creatinine on discharge, mg/dL, median (IQR)	1.1 (0.9–1.4)	1.5 (1–2.2)	0.0002
Troponin I on admission, per ng/mL, median (IQR)	9.4 (3.6–23.5)	40.5 (8.7–106.3)	<0.0001
Maximum troponin I during hospital stay, ng/mL, median (IQR)	11 (4–29.8)	79 (11.1–578.9)	<0.0001
Hemoglobin on admission, g/dL, median (IQR)	7.1 (6.3–7.8)	7.1 (6.2–8)	0.8532
MCV on admission, fL, median (IQR)	75.7 (66–86.8)	85.7 (77.4–91.4)	0.002
MCH on admission, (pg), median (IQR)	23.8 (20–28.3)	27.3 (23.8–29.7)	0.0052
MCHC on admission, g/dL, median (IQR)	31.1 (30–32.3)	31.9 (30.8–32.7)	0.0562
RDW, %, median (IQR)	19.5 (17.3–22.3)	18.4 (16.4–21.5)	0.2045
WBCs, K/μL, median (IQR)	7.1 (5.3–9.4)	8.5 (6.1–13.3)	0.0096
Platelets, K/μL, median (IQR)	254 (181.5–335.5)	235 (143–297)	0.1027
TSH, mU/L, median (IQR)	1.4 (0.8–2.1)	1.3 (0.7–1.9)	0.6211
Vitamin B12, pg/mL, median (IQR)	358 (247–566)	612 (330–832)	0.0042
Folic acid, ng/mL, median (IQR)	6.8 (4.4–11.7)	6.1 (3–14.2)	0.1997
Ferritin, μg/L, median (IQR)	21.4 (7–121.8)	165.8 (26.7–789.3)	<0.0001
Antiplatelet use, *n* (%)	232 (28.6)	10 (28.6)	1
Anticoagulant use, *n* (%)	230 (28.4)	10 (28.6)	1
Proton-pump inhibitor, *n* (%)	359 (44.4)	18 (51.4)	0.488
Patients transfused, *n* (%)	686 (84.7)	29 (85.9)	0.8101
Number of PRBCs per person transfused, median (IQR)	2 (1–3)	3 (1–3)	0.0084
Gastroscopy performed, *n* (%)	454 (56)	11 (31.4)	0.005
Colonoscopy performed, *n* (%)	331 (40.8)	6 (17.1)	0.0044
Diagnosis of anemia			
Multifactorial, *n* (%)	398 (49.1)	21 (60)	0.2298
Iron deficiency, *n* (%)	481 (59.3)	12 (32.3)	0.0045
Vitamin B12 deficiency, *n* (%)	115 (14.2)	4 (11.4)	0.8066
Folate deficiency, *n* (%)	26 (3.2)	2 (5.7)	0.3242
Hematologic disease, *n* (%)	87 (10.7)	6 (17.1)	0.2627
Gastrointestinal loss **, *n* (%)	364 (68.9)	11 (64.7)	0.7911
Urogenital blood loss, *n* (%)	58 (7.2)	3 (8.6)	0.7344
CKD-associated anemia, *n* (%)	344 (42.4)	25 (71.4)	0.0008
Hemolysis, *n* (%)	28 (3.5)	3 (8.6)	0.132
Hemoglobinopathies, *n* (%)	54 (6.7)	1 (2.9)	0.7215
Other *, *n* (%)	76 (9.4)	4 (11.4)	0.5644
Duration of stay, days, median (IQR)	4 (2–6)	5 (3–14)	0.0101

* Metastatic malignancy, cirrhosis, heart failure, hypothyroidism, drug-induced. ** Among patients where endoscopy was performed. CKD: chronic kidney disease; IQR: interquartile range; MCH: mean corpuscular hemoglobin; MCHC: mean corpuscular hemoglobin concentration; MCV: mean corpuscular volume; OR: odds ratio; RDW: red cell distribution width; TSH: thyroid stimulating hormone; WBCs: white blood cells.

**Table 4:** Regression analysis of mortality among patients admitted with anemia.

Characteristic	Univariate Analysis *p*	Multivariate Analysis *p*	OR (95% CI)
Age (per year)	0.022	0.524	1.014 (0.972–1.057)
History of CKD	0.0001	0.124	2.946 (0.744–11.665)
Troponin I on admission (per ng/mL)	<0.0001	0.199	1 (1.000–1.001)
Maximum troponin I during hospital stay (ng/mL)	<0.0001	0.503	1 (1.000–1.000)
MCH on admission (per pg)	0.0151	0.364	1.035 (0.961–1.113)
Vitamin B12 (per pg/mL)	0.0011	0.023	1.001 (1.000–1.001)
Diagnosis of iron deficiency as a cause of anemia	0.0019	0.438	0.715 (0.306–1.669)
Diagnosis of CKD as a cause of anemia	0.0004	0.469	1.504 (0.498–4.537)
Duration of stay (per day)	<0.0001	0.000	1.071 (1.032–1.113)

CI: confidence interval; CKD: chronic kidney disease; MCH: mean corpuscular hemoglobin; OR: odds ratio.

## Data Availability

The data presented in this study are available on request from the corresponding authors.
